# In Vitro and In Vivo Functional Characterization of Essence of Chicken as An Ergogenic Aid

**DOI:** 10.3390/nu10121943

**Published:** 2018-12-07

**Authors:** Shih-Wei Huang, Yi-Ju Hsu, Mon-Chien Lee, Hua-Shuai Li, Paul Chee Wei Yeo, Ai Lin Lim, Chi-Chang Huang

**Affiliations:** 1 Graduate Institute of Sports Science, National Taiwan Sports University, Taoyuan City 33301, Taiwan; 1051301@ntsu.edu.tw (S.-W.H.); 1041302@ntsu.edu.tw (Y.-J.H.); 1061304@ntsu.edu.tw (M.-C.L.); 1051304@ntsu.edu.tw (H.-S.L.); 2 Department of Physical Medicine and Rehabilitation, Shuang Ho Hospital, Taipei Medical University, New Taipei City 23561, Taiwan; 3 Department of Physical Medicine and Rehabilitation, School of Medicine, College of Medicine, Taipei Medical University, Taipei City 11031, Taiwan; 4 Department Scientific Research and Applications, BRAND’S Suntory, Singapore 048423, Singapore.; paul.yeo@brands-suntory.com; 5 Graduate Institute of Metabolism and Obesity Sciences, Taipei Medical University, Taipei City 11031, Taiwan

**Keywords:** C2C12, anti-fatigue, anti-oxidant, exercise performance, endurance

## Abstract

Essence of chicken is a popular Asian nutritional supplement that is often taken to improve metabolism and general health. Although used as a traditional remedy for combating fatigue and general health, there has been few studies investigating the ergogenic properties of chicken essence and its associated mechanism. We conducted a study to investigate the anti-fatigue and anti-oxidant properties of essence of chicken (EC) after exercise. Six weeks old male Institute of Cancer Research (ICR) mice were divided to four groups (10 mice/group) and were provided different doses of Essence of Chicken (EC): (1) Vehicle (water), (2) EC-0.5X (558 mg/kg), (3) EC-1X (1117 mg/kg), and (4) EC-2X (2234 mg/kg). EC supplementation could improve endurance and grip strength (*p* < 0.0001) and it had significant effects on the fatigue-related biochemical markers: ammonia, blood urea nitrogen (BUN), and creatine kinase (CK) levels were significantly lowered, while glucose blood levels and lactate clearance were improved after exercise challenge. Muscle and liver glycogen levels, muscle and liver superoxide dismutase (SOD), hepatic catalase (CAT), and glutathione (GSH) levels were observed to increase with EC supplementation. Preliminary in vitro data suggests that EC may have a beneficial effect in muscle mass and strength. No abnormalities were observed from pathohistological examination. Our study suggests that the EC could significantly improve exercise performance and endurance capacity and that the anti-oxidant properties of EC may be an important contributing factor to its anti-fatigue effects.

## 1. Introduction

Essence of chicken is a liquid nutritional supplement that is commonly used in Southeast Asia or Chinese medicine for various health benefits and remedies. It is consumed by the young and old and readily available in convenient packaging in several Southeast Asia countries. Made from high temperature and high pressure extraction of whole chickens, it is prevalently consumed to improve immunity, performance, metabolism, cognition, and general health (reviewed in [1]). Previous human studies have reported multiple beneficial effects of Essence of Chicken (EC) on physical health, including increase in metabolic rate [2,3], reduction in blood glucose levels and glycemic response [4,5], reduction in anxiety and depression, recovery from physical exhaustion [6], reduction in anxiety and depression [7,8], recovery from mental fatigue [9,10], and improvement in cognition [9,10,11].

EC is produced via a water extraction process from chicken meat that iscooked for several hours under high temperature, followed by centrifugation to remove fat and cholesterol, vacuum concentrated, and sterilized by high temperature and pressure before bottling. It is low in sugar and fat, rich in protein, and conveniently packaged for easy consumption and storage. EC has been shown to contain several indispensable amino acids, including threonine, methionine, lysine, tryptophan, phenylalanine, and branched chain amino acids (isoleucine, leucine, valine). In addition, several peptides, such as carnosine and anserine, minerals, trace elements, and vitamins [11,12] have also been detected. The underlying mechanism for the pleiotropic effects of chicken of essence is complicated and possibly mediated by the combined action of one or many of these bioactive components [1]. These bioactivity properties are likely to be conferred by the proteins, peptides, and/or amino acids.

In traditional Chinese medicine, some of the uses of EC are to preserve health, increase vigor, and to counter fatigue. Fatigue is a complex physiological phenomenon, which is used to describe a decrease in physical performance tht is associated with an increase in the real/perceived difficulty of a task or exercise, and it has both physical and mental aspects. The few reports on the effect of EC on fatigue have mainly concentrated on mental fatigue [9,10] with a lack of studies addressing the effect of chicken essence supplementation on exercise performance and physical fatigue. The limited studies on physical fatigue have shown somewhat heterogeneous results. Migita et al. [13] investigated the effect of EC on exercise-induced physical and mental stress reduction during intensive long distance training in male runners. They saw that, even though EC ingestion was able to reduce the mental stress during training, EC did not appear to aid physical fatigue recovery, as assessed by anemic index, lipid peroxidation, and the serum levels enzymes for muscle tissue damage. More recently, a human study by Lo and others [6] showed that EC supplementation given after a single bout of exhaustive exercise was able to reduce the post-exercise accumulation of lactate and ammonia in the plasma. However, levels of plasma glucose, creatine kinase, and heart rate during the post-exercise recovery phase were no different between the EC and placebo supplementation groups.

Oxidative stress is widely thought to play an important role in the etiology of fatigue, especially muscle fatigue [14]. Studies have identified reactive oxygen species production contributing to fatigue of skeletal muscles during exercise [15]. Fatigue can be delayed by antioxidant administration and it has become increasingly important to obtain efficient natural antioxidants that can exert anti-fatigue effects without any detriment or health concerns to the consumer. Sports professionals and athletes are progressively turning to dietary supplements or health foods possessing antioxidant and anti-fatigue functions as sources of energy for performance enhancement. One such example would be the use of dietary phytochemicals as an effective way to eliminate free radicals and increase antioxidant activity [16,17].

Although EC has been used shown to be beneficial for several conditions where oxidative stress plays a major role in development (reviewed in [1]), the possible use of EC as a natural nutritional supplement for exercise performance and to mitigate fatigue has not been examined. In this study, we investigate the effect of EC supplementation on exercise performance and on anti-fatigue and anti-oxidant activities in a mouse model. Additionally, we looked into possible function(s) of EC on muscle strength and muscle loss prevention.

## 2. Materials and Methods

### 2.1. Materials

The EC used is a commercially available preparation that is freeze-dried to a powder form and diluted accordingly in water prior to oral gavage feeding to the mice. EC is produced via a water extraction process from chicken meat for several hours under high-temperature, followed by centrifugation to remove fat and cholesterol. EC has been shown previously to be a protein product that is low in sugar and fat, but is rich in amino acids, proteins, and di-peptides, such as anserine and carnosine [1]. Table 1 below shows the nutritional composition of BRAND’S Essence of Chicken.

### 2.2. Animals and Groupings

Male Institute of Cancer Research (ICR) mice (six weeks old) were obtained from BioLASCO Taiwan Yi-Lan Breeding Center in Taiwan. The mice were fed a standard laboratory diet (No. 5001; PMI Nutrition International, Brentwood, MO, USA) and distilled water *ad libitum*. They were maintained under standard laboratory conditions of 12-h light/12-h dark cycle at room temperature (22 ± 2 °C) with 50%–60% humidity. The bedding was changed and cleaned twice per week. All of the animal experiments followed the Law for the Humane Treatment and Managements of Animals and other related laws and regulations. All the experiments were conducted under a protocol that was approved by the Institutional Animal Care and Use Committee (IACUC) ethics committee of National Taiwan Sport University and the study conformed to the guidelines of the protocol IACUC-10629.

After two weeks of acclimatization, 40 mice were randomly divided to four groups (*n* = 10/group) for once-a-day oral gavage of vehicle or EC for 28 days: Group 1 (vehicle control or water only); Group 2 (0.5X EC dose or 558.5 mg/kg); Group 3 (1X EC dose or 1117mg/kg); Group 4 (2X EC dose or 2234 mg/kg). For all tests and samples, 10 mice per group were used. The quantity of food and water consumed by each group of mice was monitored daily and the body weight of the mice was measured weekly.

In this study, the EC dose used for the mice was using the guide for dose conversion previously described [18]. Based on previous clinical data, the daily recommended dose of EC in humans is 68 mL or equivalent 5.45 g freeze-dried powder. To convert human dose to the animal dose in mice, we assume a human weight of 60 kg and body surface area correction factor/conversion coefficient of 12.3. For the 1X EC dose, the mouse dose used was 5.45 (g)/60 (kg) = 0.0908 × 12.3 (conversion coefficient) = 1117 mg/kg.

### 2.3. Sample Collection

Three days after the last experiment (90 min free swimming test), all animals were given the last dose of vehicle of EC dose and euthanized with 95% CO_2_ asphyxiation. Blood was immediately collected by cardiac puncture and serum obtained by centrifugation at 1500× *g* for 10 min at 4 °C. The liver, kidney, heart, lung, skeletal muscle (including gastrocnemius and soleus muscles in the back part of the lower legs), epididymal fat pat (EFP), and brown adipose tissue (BAT) were excised and weighed. The liver and gastrocnemius muscle were collected immediately after saline cleaning. Those samples were maintained at −80 °C until the analysis of glycogen content.

### 2.4. Weight-Loaded Forced Swimming Test (WFST)

The weight loaded forced swimming test was performed as previously described [19]. Briefly, 30 min after the last dose was administered on Day 28 of treatment, mice taken from each group were subjected to the force swimming exercise. Each animal was supplied with a constant load equivalent to 5% of the body weight tagged to the tail and placed individually in a columnar swimming pool (65 cm high with 20 cm radius) that was filled with water to a depth of 40 cm and maintained at 27 ± 1 °C. Swimming time was recorded as from the beginning of swimming to the point of exhaustion, determined by observing loss of coordinated movements and failure to return to the surface within 10 s. The exhaustive swimming time was used as an index of exercise endurance.

### 2.5. Forelimb Group Strength

A digital force meter (Model-RX-5, Aikoh Engineering, Nagoya, Japan) was used to measure the forelimb grip strength, as previously described [3]. The force gauge was equipped with a metal bar (2 mm in diameter and 7.5 cm long) to measure the maximum force that is applied by the mouse. The mouse was lifted at the base of the tail, lowered vertically towards the bar and allowed to grasp the bar mounted on the force gauge with both forepaws. The mouse was gently pulled away at a constant speed until its grip was broken. Peak tension (grams of force) was recorded on the digital force gauge as the mouse released its grip. The maximal force (grams) exerted by the mouse was used as the forelimb grip strength. Relative grip strength was calculated as strength per gram body weight. Each mouse was subjected to the grip strength test 30 min after the last treatment dose was administered. We performed 10 consecutive measurements in a one-minute interval for each mouse.

### 2.6. Cell culture and Treatment

C2C12 myoblasts were purchased from American Type Culture Collection (ATCC, Manassas, VA, USA) and were cultured in DulECco’s Modified Eagle’s Medium (DMEM, Sigma-Aldrich, St. Louis, MO, USA) supplemented with 20% (*v*/*v*) fetal bovine serum (FBS, Lonza, Walkersville, MD, USA) and 1% penicillin-streptomycin. C2C12 myoblasts were maintained at 37 °C in a humidified 5% CO_2_ incubator. When the myoblasts reached 80% confluence, they were differentiated in to myotubes in DMEM containing 2% horse serum (Thermo Fisher Scientific, Inc., Waltham, MA, USA), with medium change every two days. Differentiation was complete in 5–7 days, and the myotubes remained viable for a further 4–5 days. Differentiated myotubes were treated for 72 h with 100 ng/mL tumor necrosis factor alpha (TNF-α, Sigma Aldrich, St. Louis, MO, USA) or 12.5 mg/mL EC or both compounds in combination. Myotubes were photographed at 10× magnification using the 1X71 inverted microscope (Olympus, Tokyo, Japan) and measured using the CellSens software (Olympus, Tokyo, Japan). At least three independent fields were taken for each treatment group and diameter of 15 myotubes measured in each field.

### 2.7. Blood Biochemical Indices upon Acute 10-min Free Swimming Test

The effects of EC on plasma lactate, ammonia, and glucose levels were evaluated after four weeks of administration. One hour after the EC dose was administered, the mice were subjected to a 10-min swimming test without weight-loading. The swimming pool (65 cm high with 20 cm radius) was filled with water to a depth of 40 cm and maintained at 27 ± 1 °C. Blood samples were collected before and after the swimming exercise by submandibular blood collection. Serum lactate and glucose levels were measured at three time points: 1) before exercise, 2) after 10-min swimming test, and 3) 20 min of rest after swimming. Ammonia levels were obtained before and immediately after swim test. At all time points, 50–80 µL blood was collected from each mouse. Total blood collection is controlled within 10% of the mouse body weight. Serum was collected by centrifugation at 1500× *g* and 4 °C for 10 min. Lactate, ammonia, creatine kinase (CK), and glucose levels were determined by use of an autoanalyzer (Hitachi 7060, Hitachi, Tokyo, Japan).

### 2.8. The 90-min Free Swimming Test with Serum Biochemical Measurements

Two days after the 10-min free swimming test, mice from each group were subjected to a 90-min free swimming challenge without a weight load. The swimming pool (65 cm high with 20 cm radius) filled with water to a depth of 40 cm and maintained at 27 ± 1 °C. At the end of the swim, the mice rested for one hour before blood samples were taken for urea nitrogen analysis (BUN) and creatine kinase (CK) analysis. Serum (50–80 μL) was collected from each mouse, with total blood collection controlled within 10% of the mouse body weight. Serum was obtained by centrifugation at 1500× *g* for 10 min at 4 °C. Serum levels of clinical biochemical variables, including aspartate aminotransferase (AST), alanine aminotransferase (ALT), creatine kinase (CK), total protein (TP), albumin, blood urea nitrogen (BUN), creatinine, uric acid (UA), total cholesterol (TC), and triacylglycerol (TG) were measured with an autoanalyzer (Hitachi 7060, Hitachi, Tokyo, Japan).

### 2.9. Measurement of Antioxidant Status in Liver and Muscle

The antioxidant status of the liver and muscle tissues were measured commercial assay kits from Cayman Chemical (Ann Arbor, MI, USA), according to the manufacturer’s protocol. For the superoxide dismutase (SOD) assay kit, all three types of SOD (Cu/Zn, Mn, and Fe SOD) were measured and utilized a tetrazolium salt for the detection of superoxide radicals generated by xanthine oxidase and hypoxanthine. One unit of SOD was defined as the amount of enzyme that is needed to produce 50% dismutation of superoxide radical.

Glutathione peroxidase (GPX) activity was measured using the glutathione peroxidase assay kit (Cayman chemical, Ann Arbor, MI, USA), according to manufacturer’s instructions. The measurement of GPX activity is based on the principle of a coupled reaction with glutathione reductase (GR). The oxidized glutathione formed after the reduction of hydroperoxide by GPX is recycled to its reduced state by GR in the presence of nicotinamide adenine dinucleotide phosphate (NADPH). The oxidation of NADPH is accompanied by a decrease in absorbance at 340 nm. One unit of GPX was defined as the amount of enzyme that catalyzes the oxidation of 1 nmol of NADPH per minute at 25 °C. Glutathione reductase (GR) is a flavoprotein that catalyzes the NADPH-dependent reduction of oxidized glutathione (GSSG) to reduced glutathione (GSH). For GR determination, tissue was homogenized in cold buffer (50 mM potassium phosphate, pH 7.5, 1 mM EDTA) and centrifuged at 10,000× *g* for 15 min (4 °C). The resulting supernatant was used to measure GR activity by measuring the rate of NADPH oxidation. The oxidation of NADPH to NADP^+^ is accompanied by a decrease in absorbance at 340 nm.

Catalase (CAT) activity was measured using the assay kit (Cayman chemical, Ann Arbor, MI, USA), which utilizes the peroxidase function of catalase to determine enzyme activity. The method is based on the reaction of the enzyme with methanol in the presence of an optimal concentration of H_2_O_2_. For Glutathione (GSH) activity, the tissues were homogenized with 0.1 M sodium phosphate buffer (pH 7.4) and the homogenates subsequently centrifuged with metaphosphoric acid (MPA) solution to remove the proteins. A 50-μL aliquot of the homogenate was mixed with 150 μL reaction buffer (provided in the kit), vortexed, and the absorbance at 405 nm taken within 30 min. The glutathione content was calculated using a standard solution of glutathione. Total protein concentrations of samples were determined using a DC protein assay kit (Bio-Rad Laboratories, Hercules, CA, USA).

### 2.10. Tissue Glycogen Concentration

After mice were sacrificed, the muscles and liver were excised and weighed for subsequent glycogen content analysis. The method of glycogen analysis used was as described previously [20]. Briefly, for each mouse, 100 mg of liver and muscle tissue was finely cut, weighted, and homogenized in 0.5 cold perchloric acid. After centrifugation for 15 min at 15,000× *g* at 4 °C, supernatant was discarded. Standard glycogen (Sigma-Aldrich, St. Louis, MO, USA) or tissue extracts (30 µL) were added to wells of a 96-well plate, followed by an iodine-potassium iodide reagent (200 µL). Plate was allowed to rest for 10 min before measuring the absorbance at 460 nm by an ELISA reader (Tecan Infinite M200, Tecan Austria, Austria).

### 2.11. Histological Staining of Tissues

Liver, skeletal muscle, heart, lung, kidney, epididymal fat pat (EFP), and brown adipose tissue (BAT) were harvested and fixed in 10% formalin after the mice were sacrificed. Following formalin fixation, tissues were embedded in paraffin and were cut into 4-μm thick slices for morphological and pathological evaluations. Tissue sections were stained with hematoxylin and eosin (H&E) and examined by light microscopy with a CCD camera (BX-51, Olympus, Japan) by a clinical pathologist.

### 2.12. Statistical Analyses

Data are expressed as mean ± SEM (*n* = 10) and significance is set at *p <* 0.05. Statistical analysis was done by using one-way analysis of variance (ANOVA) followed by Duncan’s post-hoc test for multiple comparisons. All analyses were performed using SAS version 9.4 (SAS Inst., Cary, NC, USA) with Relationships between clinical variables and dose–response trend were calculated by Pearson’s-correlation coefficients using the CORR procedure. For in vitro analysis, one-way ANOVA was used followed by Tukey’s post hoc test. *p* < 0.05 was considered to be statistically significant.

## 3. Results

### 3.1. Effect of EC on Food and Water Consumption, Body Weight and Organ Weight

Administration of EC at different doses over 28 days did not bring about significant differences in food and water intake (Table 2). Additionally, there was no significant differences in final body weights (40.4–41.0 g), as well as absolute or relative weights of organs (liver, kidney, heart, lung, muscle, EFP, or BAT) between the EC groups with vehicle control group. Relative organ weight was calculated by taking the absolute weight divided by the mouse weight and converted to a percentage.

### 3.2. Effect of Four-Week EC Supplementation on Weight Loaded Forced Swimming Test

**Figure 1:**
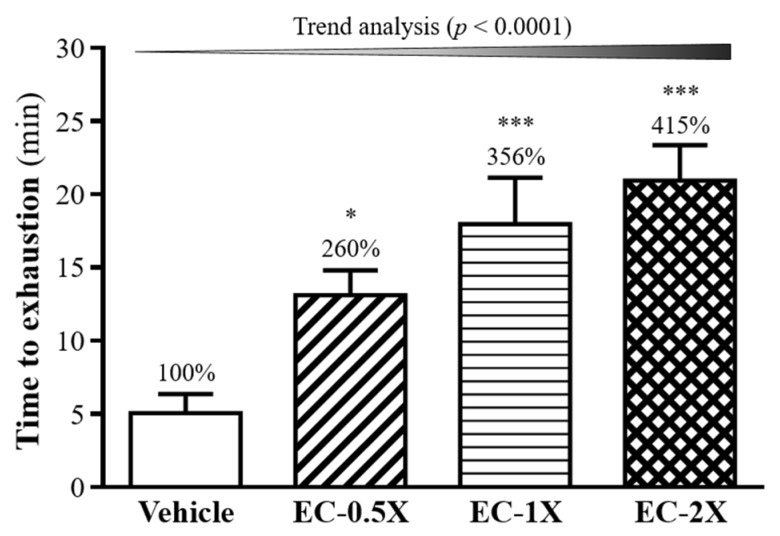
Effect of a four-week EC supplementation on endurance swimming performance in mice. Mice were pretreated with the vehicle, EC-0.5X, EC-1X, and EC-2X for 28 days. Data are expressed as mean ± SEM (*n* = 10). Numbers indicate percentage (%) change with vehicle group fixed at 100%. Significant difference from control group according to one-way ANOVA is indicated * *p <* 0.05, *** *p <* 0.001.

As shown in Figure 1, the time to exhaustion of each EC group was significantly longer (*p <* 0.05) than that recorded for the vehicle control group. The maximum forced swimming times were 13.10 ± 1.71, 17.95 ± 3.18 and 20.93 ± 2.42 min for the EC-0.5X, EC-1X, and EC-2X groups, respectively. When compared with the control group’s exhaustive time of 5.05 ± 1.30 min, EC was able to enhance the swimming capability by delaying the onset of physical fatigue in the mouse by 260%, 350%, and 415% with an increase in EC dose from 0.5X, 1X, to 2X, respectively. The enhancement in swimming capacity and endurance was shown to be dose-dependent (*p <* 0.0001).

### 3.3. Effect of EC Supplementation on Forelimb Grip Strength

**Figure 2:**
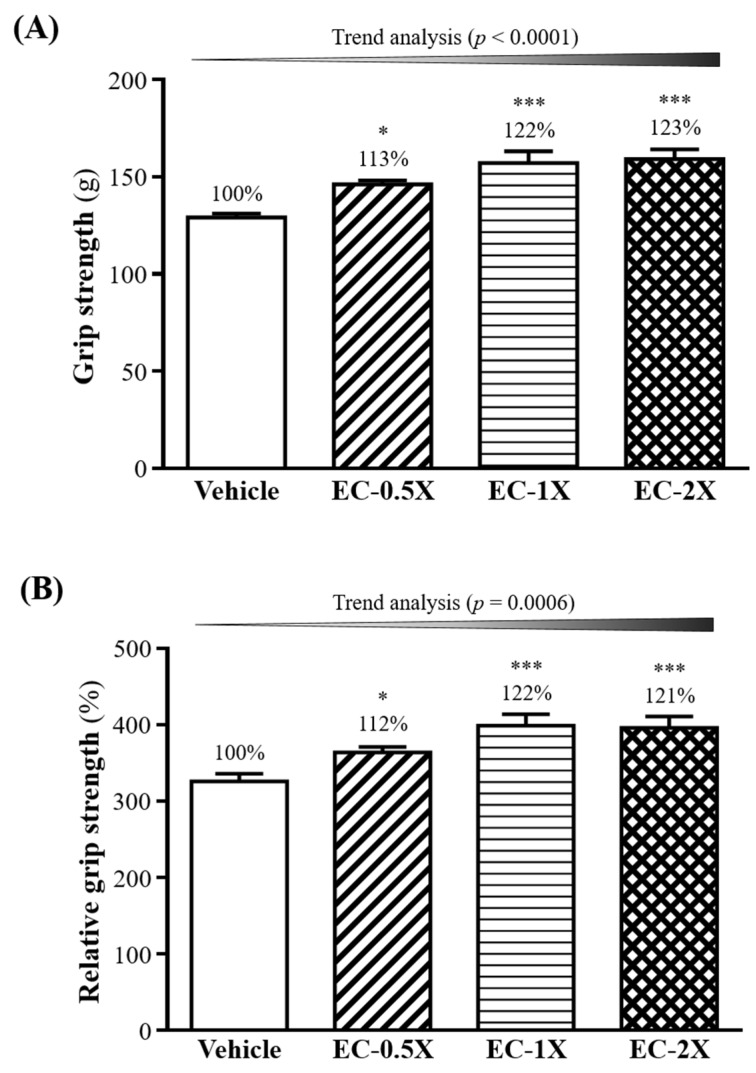
Effect of a four-week EC supplementation on: (**A**) forelimb grip strength and (**B**) relative forelimb grip strength in mice. Mice were pretreated with the Vehicle, EC-0.5X, EC-1X, and EC-2X for 28 days. Data is expressed as mean ± SEM (*n* = 10). Numbers indicate percentage (%) change with vehicle group fixed at 100%. Significant difference from control group according to one-way ANOVA is indicated * *p <* 0.05, *** *p <* 0.001.

In general, programed exercise training is required to improve grip strength [21]. However, we found that EC supplementation for four weeks was able to improve forelimb grip strength, even though the tested mice did not undergo any training intervention. Figure 2A shows that the grip strength was significantly higher with the EC-0.5X (146 ± 2 g), EC-1X (157 ± 6 g) and EC-2X (159 ± 5 g) groups than the control group (129 ± 2 g). This represented an increase in grip strength of 13% (*p <* 0.0072), 22% (*p <* 0.0001), and 23% (*p <* 0.0001) for the EC-0.5X, EC-1X, and EC-2X groups, respectively, as compared with the control group. The effect of EC on forelimb grip strength was shown to be dose dependent (*p <* 0.0001). Similarly, the same dose-dependent effect of EC was shown by comparing the relative grip strength, which is calculated by the grip strength (g)/ body weight (g) X 100%. As shown in Figure 2B, the relative grip strength of the EC-0.5X (364 ± 7%), EC-1X (399 ± 15%), and EC-2X (396 ± 15%) groups significantly increased by 112%, 122%, and 121%, respectively, as compared to the Vehicle group (326 ± 10%).

### 3.4. Effect of EC Treatment on C2C12 Myotubes

**Figure 3:**
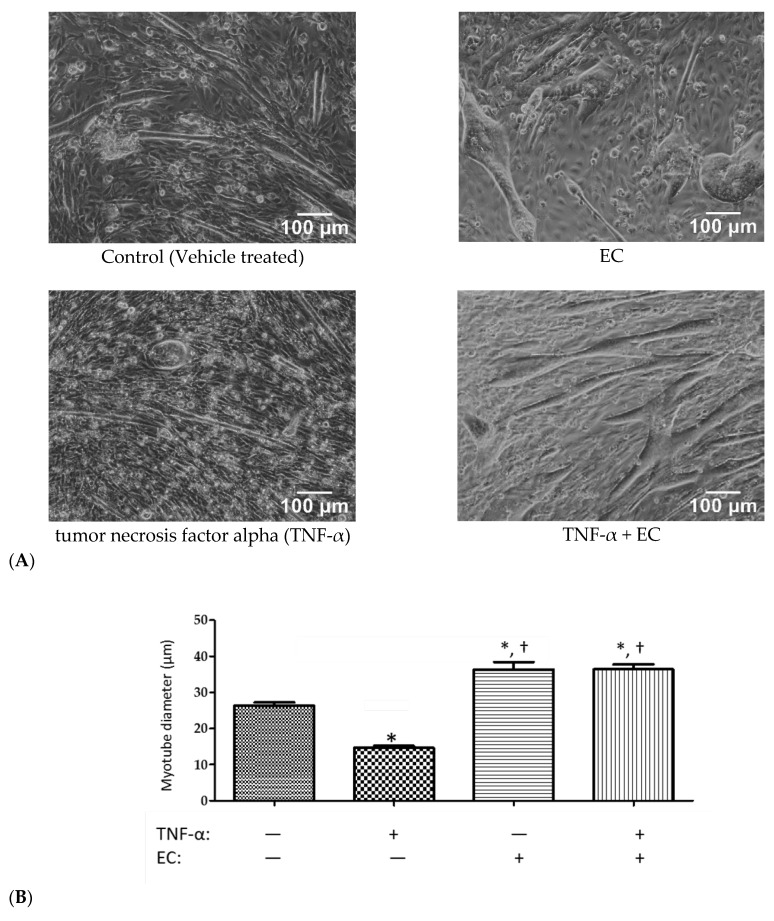
Hypertrophy effects of EC on the myotube size. C2C12 cells were induced to differentiate, followed by presence or absence of TNF-α (100 ng/mL) and/or EC (2.5 mg/mL) added together as a co-treatment. (**A**) Representative images of the C2C12 myotubes following 72 h treatment in the presence and absence of TNF-α and/or EC observed by microscopy (×100 magnification). (**B**) Measurement of average myotube diameter after 72 h treatment. Diameter was assessed by CellSens based on three independent fields per sample, with 15 myofibers randomly selected per field. Results are presented as the mean diameter ± SEM. Differences from control (without TNF-α or treatment) are shown as * *p* < 0.05, while differences from TNF-α alone are shown as † *p* < 0.05.

The effect of EC in muscles has previously not been studied, so we further investigated the effect of EC on muscle using an in vitro culture of C2C12 murine myoblasts (Figure 3). The C2C12 cells have been shown to be an adequate model to monitor skeletal muscle behaviour and identify nutrient compounds with bioactivities to promote hypertrophy or reduce atrophy [22,23]. In this study, C2C12 skeletal muscle myoblasts were induced to differentiate in mitogen-poor media, such as 2% FBS to form multinucleate myotubes, followed by 72 h treatment in the presence or absence of 2.5 mg/mL EC. Cells that were treated with TNF-α (tumor necrosis factor alpha) were able to induce muscle atrophy and cause a 44% reduction in mean myotube diameter to 14.7 µm from 26.4 µm in the control untreated group. Co-treatment with EC not only completely prevented the effects of TNF-α, but the myotubes showed a mean diameter of 36 µm that was significantly larger (*p* < 0.001) than the control cells. EC treatment alone was sufficient to significantly alter myotube diameter size, increasing the mean diameter to 36 µm, which was significantly higher (*p* < 0.0001) than the untreated control group, but insignificant from the TNF-α + EC treatment group.

### 3.5. Effect of EC Supplementation on Lactate after a 10-min Swimming Test

After four weeks of supplementation, mice underwent a 10-min swimming test to evaluate the levels of lactate before and after the exercise, as well as after resting 20 min (Table 3). Before swimming, there was no significant differences in the levels of blood lactate among the vehicle, EC-0.5X, EC-1X, and EC-2X groups. After the 10 min swimming test, the levels of blood lactate of the EC-0.5X (4.8 ± 0.1 mmol/L), EC-1X (4.8 ± 0.2 mmol/L), and EC-2X (4.3 ± 0.1 mmol/L) groups were significantly lower by 22.44% (*p <* 0.0001), 23.40% (*p* < 0.0001), and 30.77% (*p* < 0.0001), respectively, as compared with the control group (6.2 ± 0.1 mmol/L). This effect of EC post-exercise lactate levels was dose-dependent *p* < 0.0001).

After 20 min rest following the swimming test, the levels of blood lactate of the EC-0.5X (3.3 ± 0.1 mmol/L), EC-1X (3.3 ± 0.1 mmol/L), and EC-2X (3.0 ± 0.1 mmol/L) groups were all significantly lower (*p* < 0.0001). By calculating the ratio of the amounts of lactate after and before the swimming test, we demonstrated that the accumulation of lactate was significantly less by 22% (*p* = 0.0026), 23% (*p* = 0.0015) and 32% (*p <* 0.0001) in the EC-0.5X, EC-1X and EC-2X group, respectively, than the control group. Lactate clearance was also significantly (*p <* 0.05) accelerated by 50–55% with EC treatment as compared to the control group. Our results show that the EC treatment is able to suppress (22–31%) the lactate production immediately after 10 min swimming exercise and improve the removal and clearance of lactate during the recovery period (20 min rest following 10 min swimming exercise) by 50–55% as compared to control mice.

### 3.6. Effect of EC Supplementation on Serum Ammonia, Creatine Kinase and Glucose after a 10-min Swimming Test

**Figure 4:**
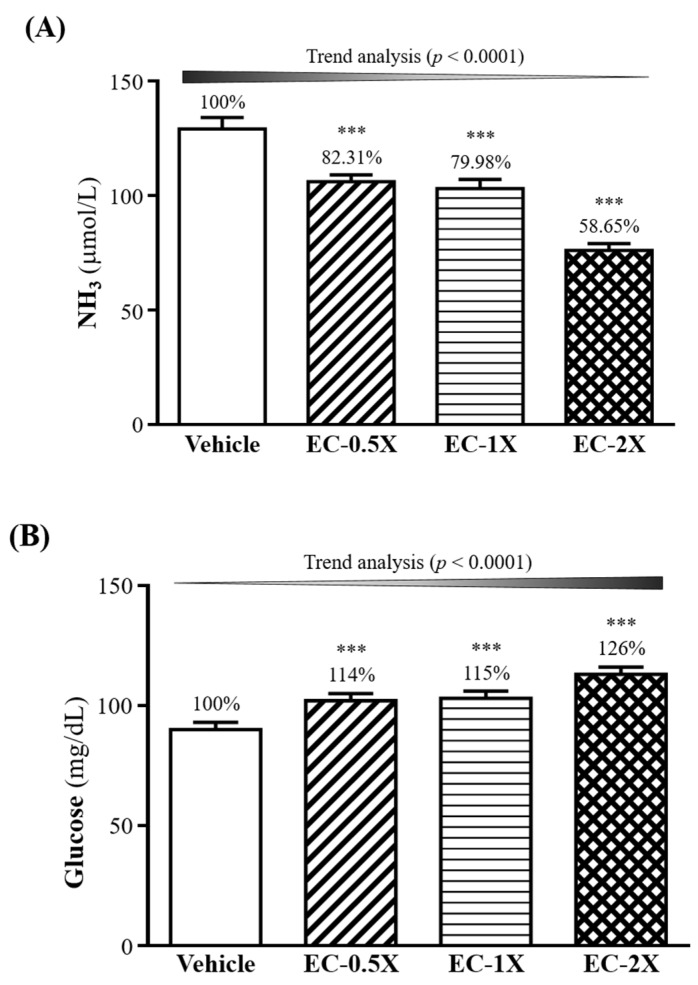
Effect of four-week EC supplementation on serum levels of (**A**) ammonia (NH_3_) (**B**) glucose after a 10-min swim test. Ammonia level was measured immediately after 10 min swim test and glucose level was measured following 20 min rest after 10 min swim test. Mice were pretreated with the vehicle, EC-0.5X, EC-1X, and EC-2X for 28 days. Data is expressed as mean ± SEM, *n* = 10. Numbers above bars indicate percentage (%) change with vehicle control set as 100%. Significant difference from control group according to one-way ANOVA is indicated *** *p <* 0.001.

After the 10-min swimming test, the serum levels of ammonia and glucose were examined (Figure 4A,B). Serum ammonia levels were lower in the EC-0.5X (106 ± 3 µmol/L, *p* = 0.0001), EC-1X (103 ± 4 µmol/L, *p* < 0.0001), and EC-2X (76 ± 3 µmol/L, *p <* 0.0001) groups than the vehicle group (129 ± 15 µmol/L) after the swimming test (Figure 4A). This corresponds to 17.7–41.35% lower ammonia levels in the EC-treated groups when compared to the control group.

For blood glucose levels that were taken immediately after the 10-min swimming test, glucose levels showed no significant difference between the EC-treated and control groups (results not shown). However, after the 10-min swim test followed by 20 min of rest, glucose levels were significantly higher (*p <* 0.0001) in the EC-0.5X (14%), EC-1X (15%), and EC-2X (26%) groups than in the control group (Figure 4B).

### 3.7. Effect of 4-Week EC Supplementation on BUN and after a 90-min Swimming Test

**Figure 5:**
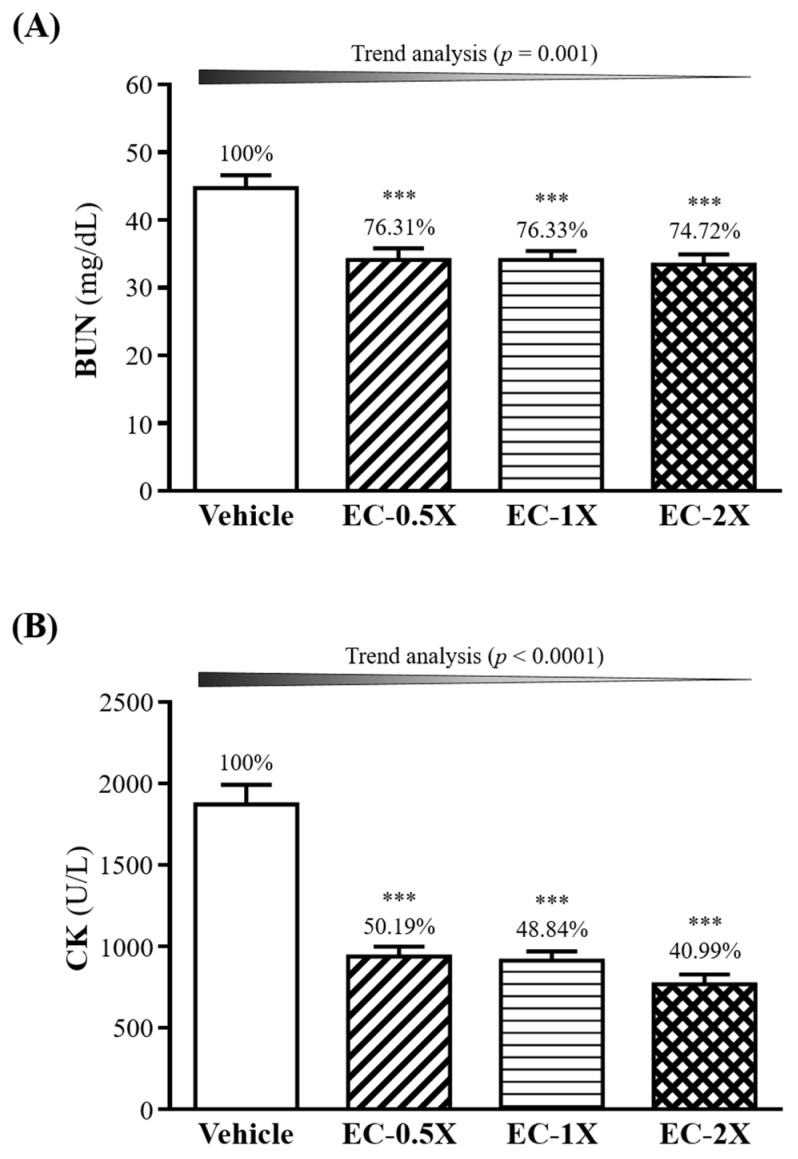
Effect of EC supplementation on (**A**) blood urea nitrogen (BUN) and (**B**) creatine kinase (CK) after 90 min swim test and 60 min rest. Mice were supplemented with the vehicle, EC-0.5X, EC-1X, and EC-2X for 28 days. Data is expressed as mean ± SEM (*n* = 10). Numbers above bars indicate percentage (%) change with vehicle control fixed at 100%. Significant difference from control group according to one-way ANOVA is indicated *** *p <* 0.001.

Serum levels of blood urea nitrogen (BUN) and creatine kinase (CK) were measured before and after a 90-min swim test (Figure 5). Levels of BUN before the swim was insignificantly different between the control (19.0 ± 0.7 mg/dL) and EC-supplemented mice (19.1 ± 0.6 mg/dL, results not shown). After the 90 min swim test followed by 60 min of rest, EC treatment showed a dose dependent effect (*p* = 0.0003) on blood urea nitrogen (BUN) levels in the mice (Figure 5A). Levels of BUN in the EC treated groups were between 33.4–34.1 mg/dL, significantly lower (*p* < 0.0001) by 24–25% as compared to that of the vehicle group (44.7 ± 6.0 mg/dL).

As shown in Figure 5B, the serum creatine kinase (CK) levels of the EC-0.5X (939 ± 60 U/L), EC-1X (914 ± 55 U/L), and EC-2X (767 ± 62 U/L) groups were significantly lower (*p <* 0.0001) by 49.81%, 51.16%, and 59.01%, respectively, as compared to the vehicle control (1872 ± 121 U/L)).

### 3.8. Effect of EC Supplementation on Liver and Muscle Glycogen

**Figure 6:**
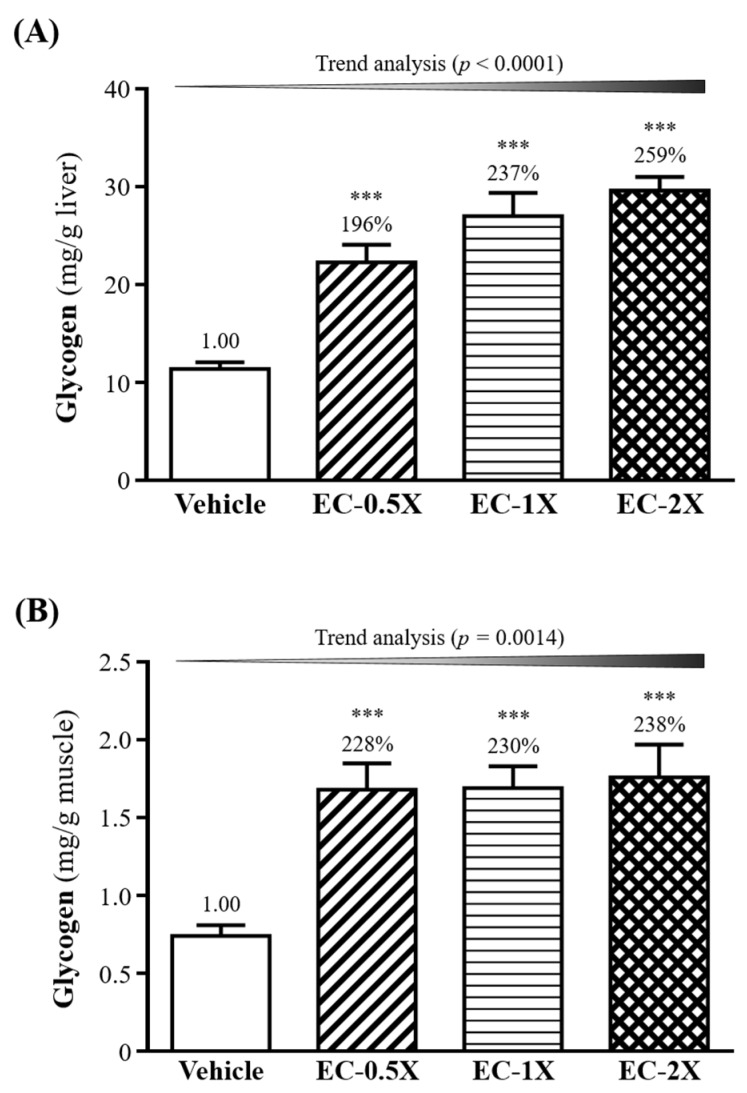
Effect of EC supplementation on glycogen levels in the (**A**) liver and (**B**) muscle. Mice were pretreated with the vehicle, EC-0.5X, EC-1X, and EC-2X for 28 days. Data is expressed as mean ± SEM (*n* = 10). Numbers above bars indicate percentage (%) change with vehicle control fixed at 100%. Significant difference from control group according to one-way ANOVA is indicated *** *p <* 0.001.

Both liver and muscle tissues were excised after the mice were sacrificed and examined for glycogen levels (Figure 6). Significantly higher and dose-dependent effects (*p* < 0.0001) were observed in both the liver and muscle tissues from the EC-treated group when compared to the control group. As shown in Figure 6A, the hepatic glycogen contents of EC-0.5X (22.3 ± 1.8 mg/g), EC-1X (27.0 ± 2.4 mg/g), and EC-2X (29.6 ± 1.4 mg/g) groups were higher by 196%, 237%, and 259%, respectively, compared to the vehicle control (11.4 ± 0.7 mg/g). Similarly, muscle glycogen levels of the EC treated groups were higher (1.68–1.76 mg/g, 228–238%) in the EC treated mice than the control group (0.74 ± 0.07 mg/g) (Figure 6B).

### 3.9. Effect of EC Supplementation on Liver and Muscle Antioxidant Status

Activity of GR in the liver and GPX, GR, CAT, or GSH in the muscle did not differ among the four groups (Table 4). Liver GPX activity only showed significantly higher activity (41%, *p* = 0.0121) in the EC-2X group, but not the EC-0.5X or EC-1X group as compared with the control group. Hepatic SOD, CAT, and GSH levels all showed significant EC dose-dependent increase in the EC-treated groups (*p* < 0.05) from the control group. Increases in the anti-oxidative activity for the EC-treated groups over the control group ranged from 33–43% (SOD), 34–66% (CAT) to 14–15% (GSH). The SOD activity of the muscle tissues was significantly higher (*p <* 0.0001) in the EC-0.5X (160%), EC-1X (162%), and EC-2X groups (167%) than the control group. This effect was shown to be EC dose-dependent (*p <* 0.0001).

### 3.10. Effect of EC Supplementation on Biochemical Variables at the End of the Experiment

We investigated whether the effects of four-week EC supplementation on grip strength and improved anti-fatigue performance was accompanied by biochemical changes in the blood (Table 5). Both aspartate aminotransferase (AST) and alanine aminotransferase (ALT), markers of liver damage, were not significantly different between the groups (*p* > 0.05). Mean levels of other biochemical indices, including creatine kinase (CK), total protein (TP), albumin, blood urea nitrogen (BUN), creatinine, uric acid (UA), total cholesterol (TC), triacylglycerol (TG), and glucose were also found to be unchanged between the groups (*p* > 0.05).

### 3.11. Effect of EC Supplementation on Histopathological Evaluation of Tissues

**Figure 7:**
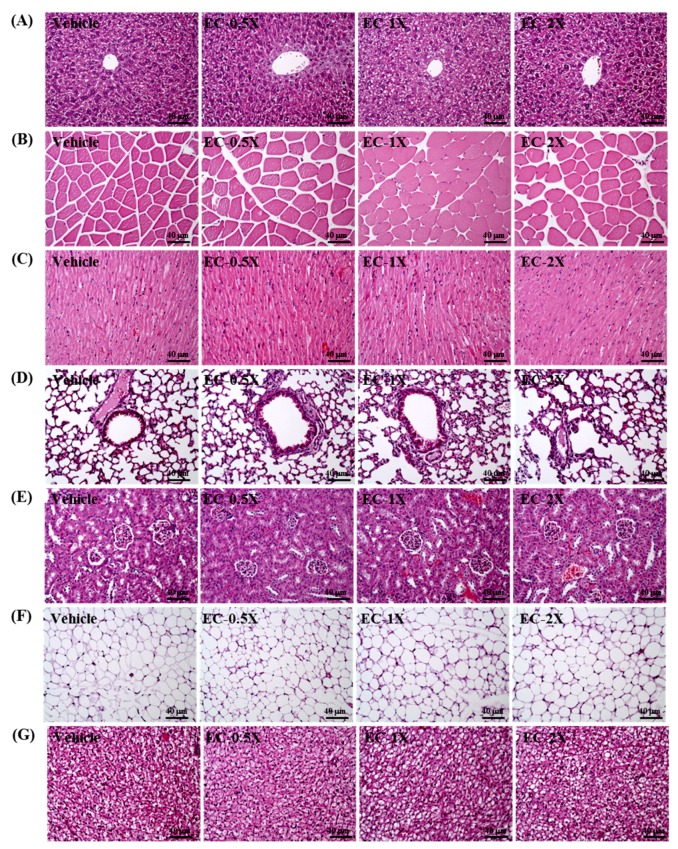
Effect of EC supplementation on histopathological evaluation of tissues, including (**A**) liver, (**B**) skeletal muscle, (**C**) heart, (**D**) lung, (**E**) kidney, (**F**) epididymal fat pad (EFP), and (**G**) brown adipose tissue (BAT). Mice were pretreated with the vehicle, EC-0.5X, EC-1X, and EC-2X for 28 days. Specimens were photographed with a light microscope (Olympus BX51). (Magnification: ×200, Scale bar, 40 µm).

EC supplementation for over four weeks had no adverse effect on major organs (Figure 7), such as the liver, skeletal muscle, heart, lung, kidney, epididymal fat pad (EFP), and brown adipose tissue (BAT). The tissues and organs examined did not show any gross abnormalities, suggesting that EC is safe for the dosage used.

## 4. Discussion

In this study, we examined the effects of EC in mice using a weight loaded forced swimming test and forelimb grip strength test. Herein, we show that supplementation of EC to the mice over a four-week period could increase the time duration of swimming to exhaustion by 216–415% in a dose-dependent manner as compared to the control mice. Even at 2X EC concentration, we had not yet detected a peak in the maximum swimming time, suggesting that the consumption of EC at a higher dose may result in greatly increased endurance and delay in fatigue onset.

Similarly, EC supplementation was able to improve another aspect of exercise performance—muscle strength, as determined by the forelimb grip strength test. In general, programmed exercise training is required to improve endurance capacity and grip strength [18]. Muscle strength may be improved via resistance training, with nutritional supplements playing a role in mitigating muscle damage or injury caused by oxidative stress or inflammation processes [24,25,26]. We found that EC supplementation for four weeks was able to improve forelimb grip strength even though the tested mice did not undergo any training intervention. Unlike the weight loaded forced swimming test that showed a strong dose-dependent increase in endurance time, the increase in muscle strength appears to be more subtle, with the effect appearing to max out at the 1X to 2X dose. This increase in muscle strength does not appear to be due to an increase in muscle mass, as there was no significant total or relative muscle mass differences between the control and EC-treated groups (Table 2). Additionally, we did not observe any significant changes in body weight or food/water intake in the mice due to EC consumption.

The effect of EC in muscles has previously not been studied, so we further investigated this small, but significant increase in forelimb muscle strength using an in vitro culture of C2C12 murine myoblasts. The C2C12 cell line is a well-established mouse myoblasts cell line used widely as an in vitro model of skeletal muscle. The mononucleated myoblasts may be differentiated in culture to form multinucleated myotube muscle fibers, with the relative thickness or diameter of these myotubes in response to treatment with atrophic or hypertrophic agents an an indicator of muscle protein synthesis or breakdown [27,28]. We showed a significant increment in diameter of EC-treated myotubes compared to control conditions. This induction of myotube hypertrophy was independent of the presence or absence of TNF-α, a mediator of the inflammatory response after injury. TNF-α induces muscle atrophy and is chronically elevated in conditions where skeletal muscle occurs, such as a result of aging, immobility, or muscle injury. Our results suggest that EC may be able to reverse skeletal muscle loss due to muscle atrophy or inflammatory conditions, promoting skeletal muscle regeneration and muscle mass regulation. This points to a possible novel role of EC in skeletal muscle hypertrophy that has not been examined to date, although further studies would be needed to determine the EC’s role in regulating skeletal muscle protein synthesis and the potential signaling pathways and associated molecular mechanisms. EC administration could represent an important strategy for reducing muscle loss due to muscle injury, catabolic diseases, or aging.

Branched chain amino acids (BCAA) make up 35% of the essential amino acids in mammalian muscle proteins and they play an important role in promoting muscle protein synthesis in vivo. Additionally, BCAAs appear to be useful nutritional supplements for sport and exercise, and for the treatment of muscle atrophy [29,30]. The abundance of BCAA in EC makes it a possible candidate for the protective effect of EC on the C2C12 myotubes. However, a recent study has shown that BCAA by itself is unable to block the atrophy induced by dexamethasone in a C2C12 model [31], suggesting that the hypertrophic effect of EC on the myotubes is likely to be due to the combination/ ratio of the amino acids or other bioactive components.

Interestingly, EC’s effect in increasing myotube size was not translated into change in muscle mass or bodyweight in the EC-treated mice, as compared to control mice. One possible explanation for these paradoxical results is that the four weeks of EC supplementation might be too short to effect significant changes in muscle mass. Additionally, the active ingredient of EC that confers skeletal muscle hypertrophy effect may not accumulate to sufficient levels in the mouse muscle, as compared with in an in-vitro system. Furthermore, resistance/strength training or an exercise regimen may be required to determine whether EC supplementation promotes any additional hypertrophic effects. This warrants further investigation, as, to our knowledge, previous human studies with EC supplementation have not measured lean body mass or muscle strength. EC is popular among the elderly consumers and any positive influence on skeletal muscle mass/strength through dietary supplementation, along with exercise intervention, could help to counteract the problems that are associated with sarcopenia and muscle loss in the elderly. Additional studies of EC supplementation with/without exercise training in an aged mouse model or human studies would help to shed light on this.

We further examined several biochemical markers that are related to fatigue in the groups of mice, including blood lactate, ammonia, and BUN. Blood lactate accumulation due to anaerobic glycolysis is seen as a response to anaerobic exercise and exercise of increasing intensity, serving as an indicator for the intensity of the exercise and the degree of fatigue. No difference in lactate levels was seen in control and EC-treated groups when the mice were at rest before exercise (Table 3). A significant increase in the concentration of lactate levels post-exercise in all of the mice indicate that the exercise was of sufficient intensity to elicit a substantial amount of skeletal muscle anaerobic glycolysis to produce energy for muscle contraction. Together with lactate, hydrogen ions are generated, presumably due to lactic acid’s dissociation. This reduction of pH in the blood and muscle tissues leads to the inhibition of muscle contraction and glycolysis, and various biochemical, harmful metabolic and physiological side effects [32]. Our results show that the EC treatment is able to suppress (22–31%) the lactate production immediately after 10 min swimming exercise and improve the removal and clearance of lactate during the recovery period (20 min rest following 10 min swimming exercise) by 50–55% as compared to control mice (Table 3). This is in agreement with the clinical study by Lo et al. [6], where essence of chicken could increase the rate of lactate removal during recovery from exercise. Levels of ammonia, another important metabolite that increases significantly with intense or prolonged exercise and is linked to physical and mental fatigue, was also found to be significantly reduced with EC supplementation in the study by Lo et al. [6] and our current study. Fatigue levels are related to increased ammonia levels during exercise [33], with excess ammonia having a toxic effect on the central nervous system. It is likely that the supplementation of EC is able to mitigate fatigue partially through decreasing the accumulation of exercise-induced ammonia.

Serum CK is known to be an indirect marker of muscle damage [34]. Serum CK level can be raised from the damage of the muscle tissue as a result of intense prolonged training and it may be a consequence of both metabolic and mechanical causes [35]. Clinically, CK levels have also been used as an indicator for several muscle-related injuries, including sarcomeric damage, muscle necrosis, muscular dystrophy, rhabdomyolysis (clinically diagnosed muscle damage), and myocardial infarction [36]. EC supplementation is able to decrease the CK activity in the blood following 90 min swim exercise, ameliorating the muscle damage resulting from exercise (Figure 5B).

Blood urea nitrogen (BUN) is an important metabolite that is formed by protein degradation after intensive exercise, with BUN levels being well correlated with the degree of exercise tolerance—the less adapted or tolerant the body is to prolonged exercise, the higher the level of BUN following protracted exercise [37,38]. The BUN test is also routinely used in clinical settings to evaluate renal function. Baseline BUN levels between the vehicle and control mice were insignificant (19.0 ± 2.3 to 19.2 ± 2.0 mg/dL). Following a 90-min free swim exercise without weights loading and a 60 min rest, control mice showed a 235% increase in BUN levels as compared to baseline (Figure 5). The accumulation of BUN indicates the speed and degree of fatigue development. EC was able to significantly attenuate the accumulation of serum BUN after exercise, suggesting that EC could enhance the body’s exercise endurance through the reduction of protein and energy metabolism.

Glycogen is an important energy source for ATP production, which is able to complement the consumption of blood glucose during exercise and help blood glucose to maintain physiological range [39]. Stored muscle and liver glycogen reserves are primary sources of energy during exercise, with muscle glycogen acting mainly as a local energy substrate for exercise and liver glycogen directly contributing to release of glucose in the blood [40]. Depletion of liver and muscle glycogen during exercise is associated with elevated fatigue, thus slower utilization of glycogen results in improved endurance capacity. Given that glycogen storage in tissues is a limiting factor of prolonged exercise, nutritional interventions that increase or maintain liver or muscle glycogen content before or during exercise could be beneficial [41,42]. The higher content of muscle and liver glycogen levels in the EC-treated groups two days post-exercise and at rest (Figure 6), despite the higher endurance of the EC-treated group suggests that EC is either able to (1) improve glycogen reserves or (2) reduce the consumption of glycogen during exercise, or both. This increase in liver and muscle glycogen reserve by EC could provide an advantage to improving the endurance performance time during prolonged exercise and mitigate any exercise-induced hypoglycemia that may be harmful to nervous function [41,43]. Given that EC treatment raised both glycogen and glucose levels (Figure 3C), it would be interesting to determine whether EC preferentially promoted the utilization of fat during prolonged exercise, a glycogen-sparing mechanism that delays the onset of fatigue.

Intense physical exercise or muscular exercise results in an increased production of radicals and other forms of reactive oxygen species (ROS), causing oxidative stress in the body. Oxidative stress produced during strenuous exercise may be responsible not only for exercise-induced fatigue, but also for impaired recovery from exercise, cell damage, or injury [43,44]. In order to minimize the risk for oxidative injury, enzymatic and nonenzymatic antioxidants work together to reduce the harmful effects of oxidants in the cells. Key antioxidant enzymes include superoxide dismutase (SOD), glutathione peroxidase (GPX), and catalase (CAT). These enzymes are responsible for removing superoxide radicals, hydroxyl radicals, and hydrogen peroxide, respectively. Nonenzymatic antioxidants include GSH, being located in aqueous compartments of the cell. Numerous animal experiments have demonstrated that the addition of antioxidants can improve muscular performance and help to fight against fatigue in sports [45]. We examined the levels of oxidative stress with indicators, including GSH, SOD, CAT, and GPX in the muscle and livers of the mice. Our results show that hepatic SOD, CAT, GSH, and muscle SOD increase significantly with increasing EC dose. GPX only significantly increased activity in the liver at the 2X EC dose. Taken together, the results suggest that the anti-fatigue effect of EC may be associated with its antioxidant activity, with EC conferring hepatic protection against exhaustive exercise-induced oxidative stress in the liver.

Our results are in agreement with previous studies demonstrating the ability of EC to protect against restraint-stress induced liver damage in mice [46] and carbon tetrachloride (CCl_4_) induced oxidative stress in rats [47]. In these studies, oxidative stress was induced and EC shown to offer protective antioxidant effects. Specifically, Zhai et al., 2012 [46] showed that the supplementation of EC was sufficient to significantly decrease MDA content (marker of oxidative damage) and upregulate the expression and activities of SOD and GPX in the plasma and liver of restraint-stressed mice. Similarly, in another model of oxidative stress by the injection of CCl4, rats that were fed with EC showed protection of liver from oxidation damage through maintaining or increasing the activities of antioxidative enzymes [47]. On the other hand, in another study of healthy rats consuming EC [48], there was no significant changes in the activity of antioxidant enzymes, possibly as a result of a balanced body condition in the absence of stress. The authors suggest that the changes in antioxidant properties afforded by EC may be more apparent with a model of oxidative stress. Herein, we show in this study that the swimming challenge is sufficient to elicit differences in antioxidant enzyme activity between the EC-supplemented and control mice, which is in agreement with these previous studies whereby the protective effect of EC was only observed under stress.

Several studies have demonstrated the usefulness of oral administration of antioxidants in reducing oxidative stress, muscle fatigue, and improving exercise performance in humans and mice models [43,45]. As EC contains a complex of bioactive peptides and amino acids with several components showing anti-oxidant activity, the effect of EC in improving exercise performance and fatigue is likely to be an overall effect of the complex rather than attributed to a single component. Many of the amino acids that are known to display antioxidant properties are found in varying degrees of abundance in EC. These amino acids include glutamic acid, aspartic acid, histidine, tryptophan, tyrosine, branched chain amino acids (BCAA—leucine, isoleucine, and valine), and other essential and non-essential amino acids. Sulfur containing compounds, such as cysteine, methionine, and taurine have antioxidative properties that are dependent on the number of sulphur atoms [49]. Histidine or histidine containing peptides have chelating and lipid radical trapping ability due to the imidazole ring [50,51], while aromatic amino acids, like tryptophan and tyrosine, are generally perceived as important contributors for the radical scavenging activity of peptides, owing to their highly reactive phenolic structures as potent hydrogen donors [52,53]. Branched chain amino acids present in EC not only have antioxidative and free radical scavenging activity [54], but have been reported to reduce muscle damage associated with endurance exercise [55]. Sun and others [56] reported that chicken hydrolysates possess notable radical scavenging activity, while Wu et al. [57] were able to identify and isolate two antioxidant peptides from a low molecular weight peptide fraction of chicken essence showing antioxidant activity. The carnosine and anserine dipeptides in EC have been determined to be potent antioxidant in skeletal muscles [58,59] and to aid in muscle contraction in skeletal tissues [60]. Additionally, the oral intake of high doses of carnosine and anserine has been found to improve high intensity and exercise performance in humans [61] and increase the swimming endurance in mice. It is likely that the bioactivity of EC is a complex interplay of the various amino acids and peptides. The mechanism and exact identification of the bioactive peptides or amino acids in EC will require further investigation.

The mice were sacrificed and examined for changes in serum biochemical markers (Table 5) or any morphological changes in the tissues and organs (Figure 7). Enzymes of liver function, such as ALT and AST, and biomarkers of renal toxicity, such as BUN and CRE, were unchanged in the control vs EC-treated mice. Furthermore, the lack of gross changes or abnormalities to the organs and tissues suggests that EC is safe for chronic consumption, providing physiological benefits without health risks or deleterious effects.

Various strategies have been employed to improve physical performance through augmenting exercise with nutritional supplements with the intent to increase muscle mass, endurance and strength. In this study, we were able to determine the ability of Essence of Chicken to improve the endurance capacity and muscle strength performance, with concomitant improvement in anti-fatigue biomarkers. The ability to mitigate exercise fatigue appears to be attributed in part to its anti-oxidative properties, which could protect cells and tissues from oxidative damage that is caused by free radicals produced during exercise. Based on our findings, we suggest that EC could be a potential ergogenic aid that is used to improve physical performance.

To date, only one clinical study on EC has looked at the effect on post-exercise recovery after a single bottle consumption of EC [6]. Our results suggest that EC supplementation itself is sufficient to improve endurance capacity and muscle strength. When used in combination with resistance or exercise training, EC supplementation may potentially exhibit additive or synergistic benefits in elevating muscle strength, adaptation, or performance in certain exercises. Further human research looking at the long term consumption of EC on performance indicators, like endurance, muscle strength, and anti-fatigue clinical parameters would help to shed light on EC’s effect on physical performance.

When compared to other protein supplements used in the market that may include whey protein, specific amino acids, creatine, or branched chain amino acids, EC provides a more holistic approach, as it is rich in many amino acids, creatine, anti-oxidants, such as anserine and carnosine, as well as potential bioactive peptides. Additionally, EC has been sold commercially for >40 years with no detrimental side effects. Consumption of EC is able to provide a wide plethora of effects, including increasing metabolic rate, reducing blood glucose levels, reducing anxiety and depression, improving physical and mental exhaustion, as well as improving cognition (reviewed in [1]), all of which would contribute to the performance of athletes. The antioxidant activity of EC warrants further elucidation and may point to new unexplored health benefits, as suggested by previous studies where dietary antioxidants, such as phytochemicals, have shown protective effects by regulating antioxidant status [16,17,43]. Further investigation would help to provide insight into the mechanism of action and to identify the bioactive peptides and amino acids that are responsible for these effects of EC.

## Figures and Tables

**Table 1:** Nutritional Content of BRAND’S Essence of Chicken (EC).

Ingredients	Amount
**Proteins and amino acids (mg/mL)**	
Protein and peptides	83.0
Free amino acid	3.1
l-anserine	2.3
l-carnosine	0.8
Taurine	0.7
**Carbohydrate (mg/mL)**	
Hexose	0.8
**Lipid (mg/mL)**	
Lipids	0.4
**Minerals (µg/mL)**	
Calcium	26
Iron	1
Zinc	2
Magnesium	32
Potassium	1740
Sodium	550
Chlorine	1340
Phosphorus	480
Sulfur	500
Copper	2
Manganese	5
Selenium	0.05
**Vitamins (µg/mL)**	
Vitamin B2	1.0
Vitamin B6	0.37
Vitamin B12	0.002
Niacin	6.4
Folic acid	0.15
Vitamin C	15

**Table 2:** General characteristics of mice with EC supplementation.

Characteristic	Vehicle	EC-0.5X	EC-1X	EC-2X	Trend Analysis
Initial BW (g)	37.30 ± 1.15	37.76 ± 0.60	37.37 ± 0.99	37.58 ± 0.90	0.9082
1st week BW (g)	38.61 ± 1.07	38.55 ± 0.86	38.43 ± 0.99	38.86 ± 0.86	0.8380
2nd week BW (g)	38.76 ± 1.08	38.84 ± 0.86	38.62 ± 1.01	39.25 ± 0.99	0.7187
3rd week BW (g)	39.58 ± 0.98	39.68 ± 0.75	39.13 ± 1.10	39.84 ± 0.90	0.8847
4th week BW (g)	39.81 ± 0.96	40.05 ± 0.70	39.64 ± 1.07	40.36 ± 0.98	0.7102
5th week BW (g)	40.14 ± 0.96	40.34 ± 0.70	40.06 ± 1.05	40.60 ± 1.99	0.7501
Final BW (g)	40.41 ± 0.86	41.58 ± 0.78	41.04 ± 1.01	40.95 ± 1.02	0.8552
Food intake (g/day)	8.95 ± 0.18	8.75 ± 0.19	8.72 ± 0.16	8.55 ± 0.14	0.1054
Water intake (mL/day)	11.02 ± 0.11	11.00 ± 0.16	11.03 ± 0.14	11.03 ± 0.16	0.9389
Liver (g)	2.310 ± 0.047	2.328 ± 0.056	2.327 ± 0.053	2.331 ± 0.081	0.8289
Kidney (g)	0.661 ± 0.021	0.663 ± 0.027	0.662 ± 0.018	0.653 ± 0.019	0.7564
Heart (g)	0.227 ± 0.012	0.229 ± 0.010	0.229 ± 0.011	0.230 ± 0.007	0.8496
Lung (g)	0.232 ± 0.007	0.232 ± 0.006	0.238 ± 0.007	0.230 ± 0.012	0.8190
Muscle (g)	0.371 ± 0.009	0.382 ± 0.007	0.379 ± 0.008	0.375 ± 0.009	0.8743
EFP (g)	0.261 ± 0.020	0.264 ± 0.029	0.262 ± 0.016	0.266 ± 0.011	0.6049
BAT (g)	0.095 ± 0.011	0.104 ± 0.010	0.101 ± 0.009	0.104 ± 0.009	0.9125
Relative liver weight (%)	5.719 ± 0.052	5.698 ± 0.081	5.672 ± 0.050	5.688 ± 0.114	0.9683
Relative kidney weight (%)	1.635 ± 0.036	1.592 ± 0.043	1.615 ± 0.027	1.594 ± 0.028	0.5164
Relative Heart weight (%)	0.562 ± 0.030	0.553 ± 0.024	0.561 ± 0.026	0.563 ± 0.020	0.8790
Relative Lung weight (%)	0.575 ± 0.015	0.559 ± 0.006	0.557 ± 0.013	0.566 ± 0.030	0.8130
Relative Muscle weight (%)	0.918 ± 0.013	0.919 ± 0.011	0.925 ± 0.013	0.916 ± 0.018	0.9830
Relative EFP weight (%)	0.646 ± 0.048	0.641 ± 0.078	0.634 ± 0.025	0.647 ± 0.019	0.5667
Relative BAT weight (%)	0.231 ± 0.023	0.252 ± 0.025	0.245 ± 0.019	0.253 ± 0.021	0.9317

Data is expressed as mean ± SEM (*n* = 10). No significant difference was detected by one-way ANOVA (*p <* 0.05). Muscle mass includes both gastrocnemius and soleus muscles in the lower legs. BAT: brown adipose tissue; EFP: epididymal fat pad. Mice were supplemented with vehicle, EC-0.5X, EC-1X, or EC-2X for 4 weeks.

**Table 3:** Effect of a four-week EC supplementation on blood lactate before and after swimming exercise.

Time Point	Vehicle	EC-0.5X	EC-1X	EC-2X	Trend Analysis
	Lactate (mmol/L)				
Before swimming [A]	3.0 ± 0.2	3.0 ± 0.2	3.0 ± 0.2	3.0 ± 0.1	0.9906
After 10 min swim [B]	6.2 ± 0.1	4.8 ± 0.1 ***	4.8 ± 0.2 ***	4.3 ± 0.1 ***	<0.0001(↓)
At rest for 20 min [C]	5.0 ± 0.2	3.3 ± 0.1 ***	3.3 ± 0.1 ***	3.0 ± 0.1 ***	<0.0001(↓)
Increase ratio [B/A]	2.16 ± 0.14	1.68 ± 0.10 **	1.66 ± 0.10 **	1.47 ± 0.07 ***	0.0002(↓)
Clearance (%) [(B-C)/B]	0.20 ± 0.03	0.31 ± 0.03 **	0.30 ± 0.03 *	0.30 ± 0.02 *	0.0717 (↑)

Mice were supplemented with the vehicle, EC-0.5X, EC-1X and EC-2X for 28 days. Data is expressed as mean ± SEM (*n* = 10). The up arrows (↑) and down arrows (↓) indicate dose-dependent increase or decrease by EC supplementation. Significant difference from control group according to one-way ANOVA is indicated * *p <* 0.05, ** *p <* 0.01, *** *p <* 0.001.

**Table 4:** Effect of EC supplementation on liver and muscle antioxidant status.

Characteristic	Vehicle	EC-0.5X	EC-1X	EC-2X	Trend Analysis
***Liver tissues***					
SOD (U/mg)	2.27 ± 0.10	3.01 ± 0.12 **	3.09 ± 0.20 **	3.25 ± 0.27 ***	0.0024↑)
GPX (nmol/min/mg protein)	2.04 ± 0.24	2.13 ± 0.17	2.56 ± 0.24	2.88 ± 0.24 *	0.0048(↑)
GR (nmol/min/mg protein)	1.79 ± 0.12	2.07 ± 0.13	2.08 ± 0.24	2.14 ± 0.24	0.2522
CAT (nmol/min/mg protein)	7.28 ± 0.39	9.78 ± 0.31 ***	10.86 ± 0.33 ***	12.12 ± 0.32 ***	<0.0001(↑)
GSH (µM/mg protein)	1.57 ± 0.12 ^a^	1.78 ± 0.13 ^b^	1.81 ± 0.13 ^b^	1.81 ± 0.12 ^b^	0.0015(↑)
***Muscle tissues***					
SOD (U/mg)	63 ± 4	100 ± 5 ***	101±6 ***	105 ± 5 ***	0.0002(↑)
GPX (nmol/min/mg protein)	201 ± 11	206 ± 10	206 ± 8	207 ± 11	0.6676
GR (nmol/min/mg protein)	122 ± 5	122 ± 6	126 ± 4	129 ± 6	0.2710
CAT (nmol/min/mg protein)	24 ± 1	25 ± 1	25 ± 1	25 ± 2	0.6670
GSH (µM/mg protein)	0.18 ± 0.01	0.19 ± 0.02	0.19 ± 0.02	0.19 ± 0.02	0.8085

Mice were supplemented with the vehicle, EC-0.5X, EC-1X and EC-2X for 28 days. Data is expressed as mean ± SEM (*n =* 10). The up arrow (↑) indicates EC dose-dependent increase. Significant difference from control group according to one-way ANOVA is indicated * *p <* 0.05, ** *p <* 0.01, *** *p <* 0.001.

**Table 5:** Effect of EC on mice serum biochemical markers at rest.

Characteristic	Vehicle	EC-0.5X	EC-1X	EC-2X	Trend Analysis
AST (U/L)	72 ± 3	72 ± 3	71 ± 3	71 ± 2	0.7116
ALT (U/L)	45 ± 3	44 ± 3	45 ± 3	43 ± 3	0.6582
CK (U/L)	124 ± 7	120 ± 7	114 ± 8	111 ± 7	0.1858
TP (g/dL)	4.9 ± 0.1	5.0 ± 0.1	5.0 ± 0.0	5.0 ± 0.1	0.5979
Albumin (g/dL)	2.7 ± 0.1	2.8 ± 0.0	2.8 ± 0.0	2.8 ± 0.1	0.6893
BUN (mg/dL)	20.3 ± 0.5	20.2 ± 0.7	20.4 ± 0.3	20.5 ± 0.5	0.7425
Creatinine (mg/dL)	0.23 ± 0.01	0.23 ± 0.01	0.23 ± 0.01	0.23 ± 0.01	0.7530
UA (mg/dL)	0.9 ± 0.1	0.9 ± 0.1	0.9 ± 0.0	0.9 ± 0.1	0.8945
TC (mg/dL)	144 ± 4	144 ± 3	141 ± 4	141 ± 3	0.4556
TG (mg/dL)	148 ± 3	144 ± 3	144 ± 2	142 ± 3	0.2008
Glucose (mg/dL)	138 ± 5	144 ± 2	143 ± 2	144 ± 3	0.2959

Levels as biochemical markers detected in the serum following 28 days of vehicle, 0.5X, 1X or 2X EC treatment. Data is expressed as mean ± SEM *n* = 10 mice/group. AST, aspartate aminotransferase; ALT, alanine aminotransferase; CK, creatine kinase; TP, total protein; BUN, blood urea nitrogen; UA, uric acid; TC, total cholesterol; TG, triacylglycerol.
